# Paralysis of the Facial Nerve

**Published:** 1857-01

**Authors:** E. Gregory


### ARTICLE IX.

### *Paralysis of the Facial Nerve.* By E. GREGORY, ESQ.

*May* 6*th*, 1853.—Mrs. W.——, aged 54 years, of a weak and delicate constitution, called and requested me to examine her teeth, she at the time suffering much pain. I did so, and found the first inferior bicuspid broken, and the root remaining; also the second inferior bicuspid very much decayed (both of the left-hand side.) At her request I extracted both tooth and stump, and on leaving she expressed much pleasure at being relieved of her pain so soon. On the following morning she called to consult me about her face, paralysis of the cervico-facial nerve having set in, so as to produce distortion of the face and draw the mouth sideways. I ordered her a blister to the back of the neck, with a full dose of castor oil, and after it had ceased acting to take the ferri sesquioxydum (carbonate of iron) in one scruple doses, every four hours; pulse being 56.

8*th*.—Much the same; pulse 56, but fuller than yesterday; blister rose well. Continue powders; take beef-tea.

*9th.*—Better; pulse the same, but increasing in volume. Continue powders; nutritious diet, with port wine.

*10th.*—Improving; pulse increasing. Take castor oil, and continue the same.

*11th.*—The face recovering; pulse increasing. Continue the same.

*13th.*—Better.

*14th.*—Better.

*15th.*—A slight relapse. Take castor oil, and use the following embrocation to the face: ℞ Lin. Saponis, ℥ ss; Liq. Vol. C. C., ʒ iij; Tt. Cantharides, ʒ j. M. ft. Linimentum, bis. vel ter die utend.

*16th.*—Much better.

*17th.*—The face nearly recovered; pulse 63, full and compressible. Take the following mixture, and call again in four days: ℞ Quinæ Disulph., gr. viij; Tt. Ferri Sesquichloridi, ʒ ij; Aqua Menth. pip., ℥ viij. M. ft. capiat coch. ij larg. bis die.

*21st.*—Quite recovered her former looks. To continue mixture for a week, and call again.

*28th.*—Quite recovered, but to continue mixture for a month at least, but to take only once a day.

I have frequently seen the patient since. Has had no relapse, enjoys better health than she has for some time before the attack. In the other two cases, I have given iron in some shape or form, with nutritious diet and blisters, and in all cases have been successful.—*Ib.*

CHELTENHAM, *July* 15.

